# A Case of Hyperammonemia Associated with High Dihydropyrimidine Dehydrogenase Activity

**DOI:** 10.1155/2016/7510901

**Published:** 2016-04-19

**Authors:** Keiki Nagaharu, Kenji Ikemura, Yoshiki Yamashita, Hiroyasu Oda, Mikiya Ishihara, Yumiko Sugawara, Satoshi Tamaru, Toshiro Mizuno, Naoyuki Katayama

**Affiliations:** ^1^Department of Hematology and Oncology, Suzuka General Hospital, Yamanohana, Yasuzuka, Suzuka, Mie Prefecture 1275-53, Japan; ^2^Department of Pharmacy, Mie University Hospital, Edobashi 2-174, Tsu, Mie Prefecture 514-8507, Japan; ^3^Department of Hematology and Oncology, Mie University Graduate School of Medicine, Edobashi 2-174, Tsu, Mie Prefecture 514-8507, Japan

## Abstract

Over the past decades, 5-Fluorouracil (5-FU) has been widely used to treat several types of carcinoma, including esophageal squamous cell carcinoma. In addition to its common side effects, including diarrhea, mucositis, neutropenia, and anemia, 5-FU treatment has also been reported to cause hyperammonemia. However, the exact mechanism responsible for 5-FU-induced hyperammonemia remains unknown. We encountered an esophageal carcinoma patient who developed hyperammonemia when receiving 5-FU-containing chemotherapy but did not exhibit any of the other common adverse effects of 5-FU treatment. At the onset of hyperammonemia, laboratory tests revealed high dihydropyrimidine dehydrogenase (DPD) activity and rapid 5-FU clearance. Our findings suggested that 5-FU hypermetabolism may be one of the key mechanisms responsible for hyperammonemia during 5-FU treatment.

## 1. Introduction

In 1957, Heidelberger et al. reported the use of 5-FU as a new antitumoral drug [1], and at present, 5-FU is one of the most commonly used anticancer drugs around the world. A combination of cisplatin and 5-FU is often used for first-line chemotherapy in unresectable cases of advanced esophageal carcinoma. As is the case for other anticancer drugs, the most common side effects of 5-FU, such as diarrhea, mucositis, neutropenia, and anemia, are due to its effects on the bone marrow and gastrointestinal epithelium. These common adverse effects are observed in more than half of the patients treated with 5-FU-containing regimens [2]. On the other hand, the prevalence of 5-FU-induced hyperammonemia has been reported to range within 5.7%–7.0% [3–5]. The exact mechanism responsible for 5-FU-induced hyperammonemia remains unknown. Herein, we report a patient who developed recurrent hyperammonemia.

## 2. Case Report

A 60-year-old man presented with a 1-month history of progressively worsening discomfort during swallowing. His medical history included treated gastric cancer (5 years earlier) and emphysema. The patient reported that he had smoked approximately 20 cigarettes per day since the age of 20. Laboratory tests did not detect hepatic disorders or renal problems. Upper gastrointestinal endoscopy revealed an ulcerative lesion with elevated distinct borders in the lower esophagus, and endoscopic ultrasound detected serosal invasion. The lesion was diagnosed as a squamous cell carcinoma from a biopsy. A positron emission tomography (PET) examination confirmed lung metastasis. As a result, the patient was clinically staged as cT3N1M1 and was treated with 5-FU and cisplatin. However, his obstructive swallowing problems continued to worsen. We next administered concurrent radiotherapy as a palliative treatment. The treatment regimen (FP regimen) consisted of 5-FU at a dose of 800 mg/m^2^ on days 1–5 and cisplatin at a dose of 80 mg/m^2^ on day 1 and was repeated every 28 days. The patient did not exhibit specific adverse effects during the first course of treatment. After the completion of that, a second course of the same regimen was started. However, the patient fell unconscious 72 hours after the initiation of treatment.

On physical examination, he was unconscious (Glasgow Coma Scale: E1V3M5) and afebrile and had a pulse rate of 69 bpm and a blood pressure of 111/61 mmHg. There were no signs of mucositis. A neurological examination did not detect paralysis or abnormal reflexes. The patient's laboratory data revealed hyperammonemia, mild hyponatremia, and a high blood urea nitrogen (BUN) level. Other findings are shown in Table 1. Radiological assessments including computed tomography (CT) and magnetic resonance imaging (MRI) scans of the patient's head did not detect any apparent cause of the patient's condition. On the following day, his condition normalized with only normal saline hydration, and he did not exhibit sequelae. We subsequently diagnosed the patient with 5-FU-related hyperammonemia.

At the onset of hyperammonemia, the patient's serum 5-FU concentration during the unconscious state was significantly lower (13 ng/mL) than the normal range (500–600 ng/mL). In 5-FU metabolism, approximately 80% of infused 5-FU is degraded by DPD, the initial and rate-limiting enzyme in the catabolism of pyrimidine bases, and this process produces ammonia as the end product. Due to the rapid clearance of 5-FU, we evaluated the patient's DPD activity using the urinary dihydrouracil to uracil ratio (DHU/U).  Table 2 shows the patient's DPD activity and a high DHU/U ratio, which indicated that his 5-FU metabolism had not been suppressed.

We concluded that continuing with the FP regimen would be harmful to the patient. He was subsequently treated with taxane-based treatment, which resulted in PD. He died approximately six months after being diagnosed due to cancer progression.

## 3. Discussion

Our patient developed hyperammonemia without other adverse effects. Laboratory examination revealed a high DHU/U ratio. Although urinary DHU/U is an indirect method to assess DPD activity, his high DPD activity was supported by his undetectably low serum 5-FU concentration and the absence of most of the common adverse effects of 5-FU treatment (including diarrhea, mucositis, neutropenia, and anemia). These findings suggested that rapid metabolism of 5-FU may cause faster than normal accumulation of ammonia.

Previous studies have investigated the relationship between DPD activity and 5-FU toxicity [7–11]. Clinically severe 5-FU toxicity was reported in a family with DPD deficiency [12]. Moreover, relatively low DPD activity was reported to be a risk factor for severe cytotoxic adverse effects [7]. On the other hand, Kim et al. described a case of hyperammonemia with high DPD synthesis, similar to our case [13]. Although the etiology for acquisition of high DPD is unknown, Li et al. demonstrated that exposure to 5-FU causes resistance to 5-FU, with upregulation of DPD activity, using a human colorectal carcinoma xenograft nude mouse model [14]. In the present and previous cases, the first administration of 5-FU may have induced upregulation of DPD activity.

There are some limitations in our hypothesis. First, we were unable to assess the actual DPD activity in the liver tissue. Because it was reported that hepatic DPD activity is correlated with the urinary DHU/U ratio, we indirectly analyzed the patient's DPD activity using the urinary DHU/U ratio, as was performed in a previous study [6]. Although urine and salivary DHU/U ratios were reported to be good predictors of the adverse effect of 5-FU [7, 15], further investigation is required to establish a method for precise DPD evaluation. Second, the DPD activity before treatment was not evaluated. Thus, we were unable to identify whether the high DPD of our case was congenital or acquired. As mentioned above, the absence of hyperammonemia during his first course suggested acquired high DPD. However, previous reports also performed the same regimen without incidence of hyperammonemia [13]. This suggests that other factors may be required for development of hyperammonemia. Third, we did not evaluate genetic analysis of DPD and other enzymes in our case. Kim et al. reported genetic mutations of thymidylate synthetase which is the main target enzyme of 5-FU in their hyperammonemia cases [16]. Not only catabolic enzymes but also the target of 5-FU may play a role in hyperammonemia.

In conclusion, the present case suggested that high DPD activity may be a trigger of hyperammonemia. While this report suggested a possible mechanism for such hyperammonemia, the exact mechanism remains unknown, and further investigation on the association between hyperammonemia and DPD activity is warranted.

## Figures and Tables

**Table 1:** Laboratory findings.

	Day of onset	The following day	[Normal range]
[Peripheral blood]			
WBC	8080/*µ*L	5180/*µ*L	3500–9000/*µ*L
RBC	382 × 10^4^/*µ*L	414 × 10^4^/*µ*L	376–500 × 10^4^/*µ*L
Hb	15.3 g/dL	12.9 g/dL	11.3–15.2 g/dL
Ht	33.3%	37.0%	33.4–44.9%
MCV	87.2 fL	89.4 fL	82.7–101 fL
Plt	26.3 × 10^4^/*µ*L	25.6 × 10^4^/*µ*L	13.0–36.9 × 10^4^/*µ*L
[Coagulation test]			
APTT	31.0 sec		25.0–45.0 sec
PT	14.2 sec		13.5–15.0 sec
D-dimer	0.66 *µ*g/mL		<0.50 *µ*g/mL
[Biochemistry]			
TP	6.2 g/dL	5.6 g/dL	6.5–8.5 g/dL
Alb	3.4 g/dL	3.4 g/dL	4.1–5.3 g/dL
AST	20 IU/L	20 IU/L	10–35 IU/L
ALT	25 IU/L	23 IU/L	10–35 IU/L
LDH	182 IU/L	155 IU/L	110–225 IU/L
*γ*-GTP		50 IU/L	8–60 IU/L
T-Bil	0.6 mg/dL	0.60 mg/dL	0.2–1.3 mg/dL
Glu	101 mg/dL		80–120 mg/dL
BUN	34 mg/dL	31 mg/dL	9.6–22.0 mg/dL
Cre	0.95 mg/dL	1.09 mg/dL	<1.20 mg/dL
Na	129 mEq/L	136 mEq/L	138–145 mEq/L
K	2.6 mEq/L	3.1 mEq/L	3.4–4.7 mEq/L
Cl	93 mEq/L	97 mEq/L	99–108 mEq/L
CRP	0.47 mg/dL	0.44 mg/dL	<0.30 mg/dL
NH^3^	131 *µ*g/dL	44 *µ*g/dL	<18 *µ*g/dL
5-FU concentration	13 ng/mL	<10 ng/mL	600 ng/mL (steady state)

Laboratory findings revealed hyperammonemia and mild hyponatremia. Serum concentration of 5-FU was low.

**Table 2:** Urinary analysis of dihydrouracil and uracil.

	Dihydrouracil	Uracil	Ratio
Patient's value	5.325 *µ*g/mL	0.495 *µ*g/mL	10.75
Normal range [6]	1.7–13.1 *µ*g/mL	4–30 *µ*g/mL	0.3–0.77

Urinary DHU/U was much higher than normal. These findings supported the high activity of dihydropyrimidine dehydrogenase.

## Abbreviation

5-FU: 5-Fluorouracil
DPD: Dihydropyrimidine dehydrogenase
CT: Computed tomography
BUN: Blood urea nitrogen
MRI: Magnetic resonance imaging
DHU/U: Dihydrouracil to uracil ratio.
